# Exploring the Bone–Liver Axis: Impact of Acute Ethanol Intoxication on Post-Traumatic Liver Inflammation and Damage Following Femur Fracture

**DOI:** 10.3390/ijms26104923

**Published:** 2025-05-21

**Authors:** Jasmin Maria Bülow, Helen Rinderknecht, Nils Becker, Kernt Köhler, Alessa Wagner, Yuntao Yang, Katrin Bundkirchen, Claudia Neunaber, Borna Relja

**Affiliations:** 1 Department of Trauma, Hand, Plastic and Reconstructive Surgery, Translational and Experimental Trauma Research, Ulm University Medical Center, 89081 Ulm, Germany; jasmin.buelow@uniklinik-ulm.de (J.M.B.); helen.rinderknecht@uniklinik-ulm.de (H.R.); nils.becker@uniklinik-ulm.de (N.B.); alessa.wagner@uniklinik-ulm.de (A.W.); yuntao.yang@uniklinik-ulm.de (Y.Y.); 2 Institute of Veterinary Pathology, Justus Liebig University Giessen, 35390 Giessen, Germany; kernt.koehler@vetmed.uni-giessen.de; 3 Hannover Medical School, Department of Trauma Surgery, 30625 Hannover, Germany; bundkirchen.katrin@mh-hannover.de (K.B.); neunaber.claudia@mh-hannover.de (C.N.)

**Keywords:** polymorphonuclear leukocytes, cytokines, Wnt, NF-κB, β-catenin, monocytes, neutrophils

## Abstract

Bone fracture activates the immune system and induces inflammation crucial for fracture healing but may also affect trauma-distant organs like the liver. Acute alcohol intoxication (AAI) dysregulates immune responses and affects organ damage post-trauma. However, the bone–liver axis and alcohol’s role in this process remain poorly understood. This study explores liver inflammation and damage following fracture, with and without prior AAI. Twenty-four male C57BL/6J mice were randomly assigned to four groups (n = 6) and received either NaCl (control) or 35% ethanol via gavage. Mice underwent femur osteotomy with external fixation or sham surgery. After 24 h, liver damage was assessed using hematoxylin–eosin and activated caspase-3 staining. Liver inflammation was evaluated through CXCL1 and polymorphonuclear leukocyte (PMNL) immunostaining, cytokine gene and protein expression analyses, and immune cell profiling in the liver via flow cytometry. Western blotting assessed NF-κB and Wnt signaling. Neither fracture alone nor with AAI caused significant liver damage. However, fracture significantly increased PMNL infiltration and altered monocyte populations, effects that were amplified by AAI. The hepatic neutrophil-to-monocyte ratio significantly decreased after fracture and was absent in the fracture AAI group. CXCL1 increased post-fracture, while MCP-1 and IL-10 decreased significantly, with AAI further significantly amplifying these changes. Wnt1 and Wnt3a levels increased significantly after fracture and were further strongly elevated by AAI. AAI completely abolished fracture-induced significant β-catenin reduction and significantly increased its phosphorylation, effects that potentially involve an AAI-induced β-catenin stabilization as well as its increased degradation. NF-κB activation was significantly decreased, while A20 expression significantly increased after fracture and AAI. Fracture influences the inflammatory liver response and signaling pathways, effects which were further modulated by AAI.

## 1. Introduction

Bone fractures are a significant global health concern, with approximately 178 million new cases documented worldwide in 2019, an increase of 33.4% since 1990 [1]. They typically result from traumatic incidents such as sports injuries, accidents, or falls, and are further exacerbated by comorbidities like osteoporosis or cancer, which weaken bone integrity [2]. Fractures disrupt bone architecture, damaging blood vessels inside the bone and surrounding soft tissue [3,4], leading to the formation of a fracture hematoma and the initiation of an inflammatory cascade [4,5,6]. This early local inflammation, including recruitment of immune cells and cytokine release, is crucial for the entire bone healing process [6,7]. However, fractures also induce systemic inflammation [8,9,10], which can extend beyond the injury site and potentially damage trauma-distant organs such as the liver [10,11].

The liver plays a central role in trauma-induced immune responses by producing acute-phase proteins, complement components, cytokines, and chemokines, and harbors a large variety of immune cells [12,13,14]. In response to trauma, it also regulates key signaling pathways, including the nuclear factor kappa-light-chain-enhancer of activated B cells (NF-κB) [15,16] or Wnt signaling pathways [17]. The NF-κB signaling pathway orchestrates the inflammatory response after trauma by regulating the expression of diverse pro- and anti-inflammatory cytokines and chemokines [15,16]. In addition, it also influences cell proliferation, apoptosis, and differentiation of innate immune cells and T cells [15,16]. The Wnt signaling pathway, while crucial for embryogenesis and hepatobiliary development, also regulates liver homeostasis, repair, and metabolism in adulthood [17,18]. A key molecule that controls and regulates both signaling pathways is the deubiquitinating enzyme tumor necrosis factor alpha-induced protein 3 (TNFAIP3), also known as A20 [19,20]. A20 cleaves the K63 ubiquitin chain from target molecules and simultaneously catalyzes the binding of K48 ubiquitin chains to trigger proteasomal degradation, which constitutes a negative feedback loop.

Additionally, beyond its immune- and inflammation-regulatory role, the liver serves as the primary organ for metabolizing and eliminating various substances such as alcohol. Alcohol consumption is widespread and socially accepted in many cultures [21]. In Germany, 7.9 million people consume alcohol to an extent that poses a health risk [22]. This does not include people drinking alcohol occasionally. Alcohol consumption significantly increases the risk of injury and leads to an altered immune and metabolic response after trauma [23]. Studies indicate that patients with elevated blood alcohol levels upon hospital admission exhibit reduced leukocyte counts and lower levels of interleukin (IL)-1, IL-6, and tumor necrosis factor (TNF)-α [24,25,26]. Similarly, trauma animal models show that acute alcohol intoxication (AAI) decreases hepatic and pulmonary inflammation [27,28,29], with reduced hepatic gene expression of *Il6*, *Tnf*, and *Il1β* in animals with AAI [27,28]. Furthermore, AAI decreases systemic leukocyte activity and neutrophil infiltration into the liver [28,29], suggesting that AAI in trauma may exert a protective effect by dampening excessive inflammation, which may damage organs. However, whether this effect is beneficial or detrimental in the context of fracture healing remains unclear.

While alcohol’s impact on immune responses and liver function after trauma has been investigated, its specific effects following bone fracture remain poorly understood. To address this knowledge gap referring to the bone–liver axis, we examined the influence of AAI on liver inflammation and injury in a murine fracture model. Male C57BL/6J mice were administered either alcohol (ethanol, EtOH) or sodium chloride (NaCl, control) via gavage before undergoing femoral osteotomy with external fixation. To assess the remote organ damage after fracture and the impact of a previous AAI, liver injury and inflammation were analyzed 24 h post-fracture using flow cytometry, histology, immunohistochemistry, gene expression, and protein expression.

## 2. Results

### 2.1. Fracture and AAI Does Not Lead to Liver Damage

**Figure 1:**
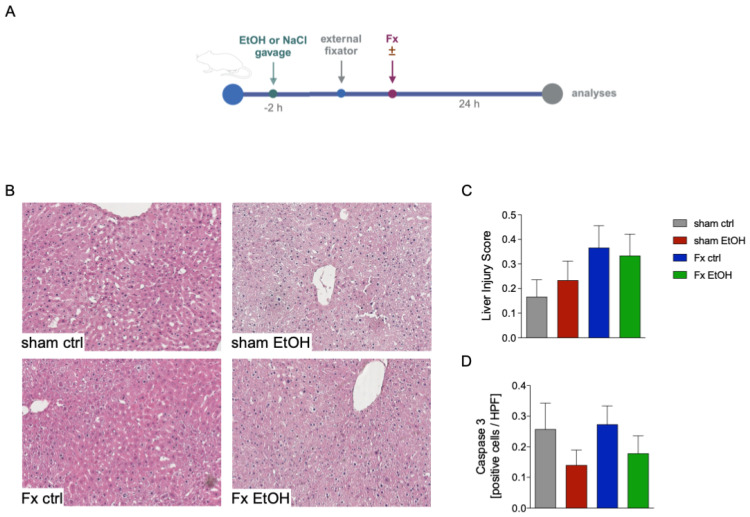
Normal liver pathology after fracture (Fx) and acute alcohol intoxication (AAI). Two hours before the initiation of experiments, the animals received an intragastric gavage of either sodium chloride (ctrl, n = 12) or ethanol (EtOH, n = 12) to simulate an AAI. Fx groups underwent osteotomy with the placement of an external fixator, and sham groups received only the external fixator. Twenty-four hours later, mice were euthanized, and sampling was performed. (**A**) Schematic illustration of the experimental setup. (**B**) Representative images of hematoxylin–eosin (HE) stained liver sections at 40× magnification. (**C**) Liver injury score and (**D**) quantitative analysis of active caspase-3 expression in the liver. Data are presented as mean ± standard error of the mean; n = 6 per group.

In this experiment, mice received either NaCl (ctrl) or EtOH gavage two hours prior to osteotomy surgery stabilized via external fixator, to mimic non-intoxication and AAI scenarios, respectively. The detailed experimental timeline is depicted in Figure 1A. To investigate the liver damage after fracture (Fx) with/without AAI, different parameters such as necrosis and vacuolization through HE staining (Figure 1B,C) were evaluated. Fracture alone (Fx ctrl) did not result in increased liver damage compared to the sham ctrl group. However, AAI slightly elevated liver injury scores in both sham-operated and fracture groups (sham EtOH, Fx EtOH, Figure 1B,C), although these increases were not statistically significant (Figure 1C).

Assessment of apoptosis via caspase-3-positive cells indicated that fracture alone (Fx ctrl) resulted in a comparable number of apoptotic cells relative to the sham ctrl group (Figure 1D). Interestingly, the two AAI groups (sham EtOH and Fx EtOH) showed slightly reduced caspase-3-positive cell numbers in the liver compared to the respective non-alcohol-treated groups, but these differences were not statistically significant (Figure 1D).

### 2.2. Increased Neutrophil Infiltration After Fracture and AAI

**Figure 2:**
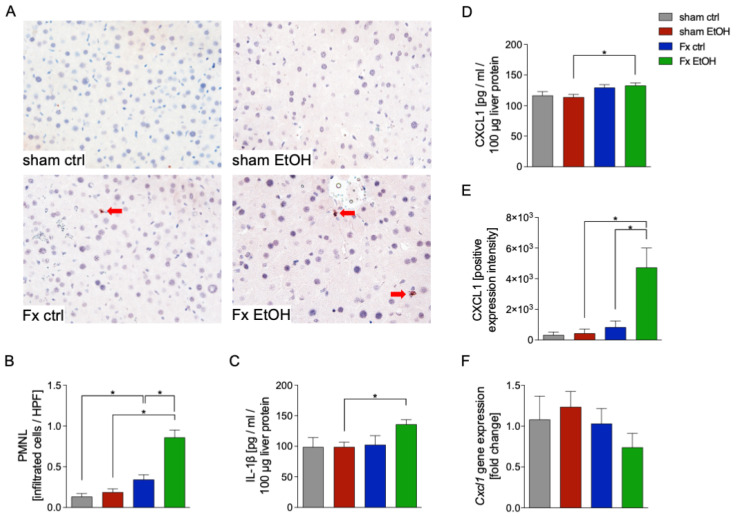
Acute alcohol intoxication (AAI) affects hepatic polymorphonuclear leukocyte (PMNL) infiltration and cytokine levels after fracture (Fx). Two hours before the initiation of experiments, the animals received an intragastric gavage of either sodium chloride (ctrl, n = 12) or ethanol (EtOH, n = 12) to simulate an AAI. Fx groups underwent osteotomy with the placement of an external fixator, and sham groups received only the external fixator. Twenty-four hours later, mice were euthanized, and sampling was performed. (**A**) Representative immune histological staining of neutrophil elastase (NE) as a marker of PMNL in liver sections at 400× magnification is represented. Red arrows indicate NE-positively stained cells. (**B**) Quantification of NE-positively stained cells per high-power field (HPF) in the liver is shown. Quantification of IL-1β (**C**) and CXCL1 (**D**) protein concentration in liver tissue homogenates using mouse-specific ELISA kits is shown. (**E**) Quantification of CXCL1-positively stained area/intensity in liver sections upon the staining of CXCL1 is shown. (**F**) RT-qPCR with homogenized liver tissue for *Cxcl1* gene expression analyses is depicted. The relative gene expression of *Cxcl1* normalized to *Gapdh* was calculated by using the comparative threshold-cycle 2^−DDCT^ method. * *p* < 0.05, n = 6 per group.

To further investigate liver damage and inflammation resulting from fracture and AAI, the infiltration of polymorphonuclear leukocytes (PMNL) via NE-staining was determined (Figure 2A,B). The results revealed that fracture alone significantly increased the infiltration of PMNL in liver tissue vs. the sham ctrl group (*p* < 0.05, Figure 2A,B). This inflammatory PMNL infiltration was further significantly enhanced by AAI in the Fx EtOH group vs. Fx ctrl group, as shown in representative immunohistochemical staining and quantitative evaluation of positively stained cells (*p* < 0.05, Figure 2A,B).

The attraction of PMNL to sites of inflammation is mediated by cytokines and chemokines such as IL-1β or CXCL1. Analysis of liver homogenates demonstrated significantly increased protein levels of IL-1β in response to fracture combined with AAI in the Fx EtOH group vs. sham EtOH group (*p* < 0.05, Figure 2C). Similarly, analysis of liver homogenates demonstrated significantly increased protein levels of CXCL1 in response to fracture combined with AAI in the Fx EtOH group vs. sham EtOH group (*p* < 0.05, Figure 2D). Immunohistochemical evaluation of CXCL1 expression intensity revealed a significant increase in the Fx EtOH group compared to both the sham EtOH and Fx ctrl group (*p* < 0.05, Figure 2E). Interestingly, gene expression of *Cxcl1* remained unchanged across all experimental groups, indicating a possible post-transcriptional regulation or differential protein stabilization as potential underlying mechanisms (Figure 2F).

### 2.3. Fracture Influences Immune Cell Proportion in the Liver

**Figure 3:**
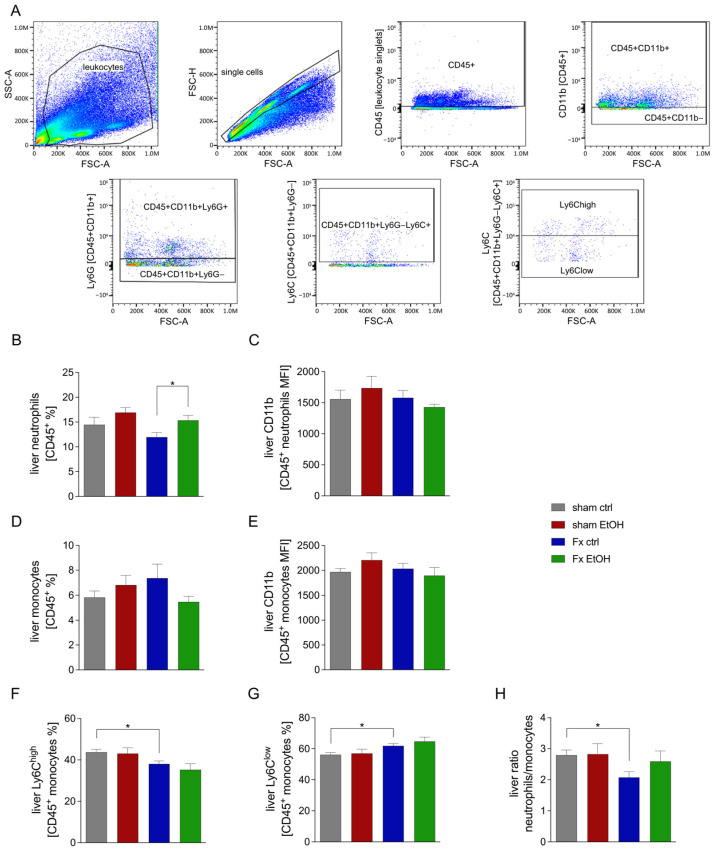
Innate immune cell proportions in the liver fracture (Fx) and acute alcohol intoxication (AAI). Two hours before the initiation of experiments, the animals received an intragastric gavage of either sodium chloride (ctrl, n = 12) or ethanol (EtOH, n = 12) to simulate an AAI. Fx groups underwent osteotomy with the placement of an external fixator, and sham groups received only the external fixator. Twenty-four hours later, mice were euthanized, and sampling was performed. (**A**) Gating strategy is shown. (**B**) Percentage of CD45+ neutrophils and their activation (**C**). (**D**) Percentage of CD45+ monocytes and their activation (**E**). Proportion of M1 monocytes (Ly6C^high^) (**F**) and M2 monocytes (Ly6C^low^) (**G**) after Fx and AAI. (**H**) Ratio of neutrophils and monocytes. * *p* < 0.05, n = 6 per group.

Immune cell proportions in liver homogenates were quantified via flow cytometry, with the applied gating strategy illustrated in Figure 3A.

The proportion of total neutrophils among leukocytes (CD45+) significantly increased in the Fx EtOH group vs. Fx ctrl group (*p* < 0.05, Figure 3B). The surface expression of CD11b, an indicator of neutrophil activation, remained unaltered by fracture or AAI (Figure 3C).

Monocytes, identified as CD11b + Ly6−Ly6C+ cells, did not show significant changes in total proportion or activation status in response to fracture alone or combined with AAI (Figure 3D,E). However, fracture, both with and without AAI, significantly reduced the proportion of the pro-inflammatory M1 monocytes (Ly6C^high^) and increased the proportion of anti-inflammatory M2 monocytes (Ly6C^low^) within the liver (Figure 3F,G).

The reduced neutrophil proportion combined with unchanged monocyte proportions resulted in a significantly lower neutrophil-to-monocytes ratio in the Fx ctrl group vs. the corresponding sham ctrl (*p* < 0.05, Figure 3H). However, this significant decline was not present in the Fx EtOH group vs. sham EtOH group (Figure 3H).

### 2.4. Fracture and AAI Influence Liver Inflammation

**Figure 4:**
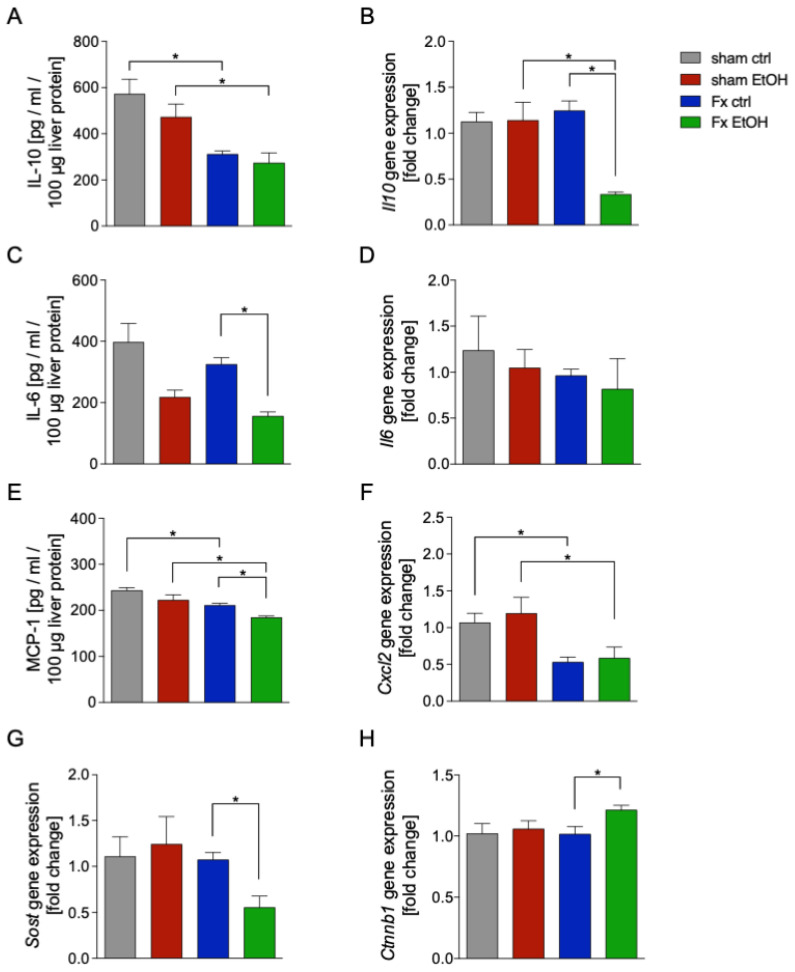
Acute alcohol intoxication (AAI) modulates liver inflammation after fracture (Fx). Two hours before the initiation of experiments, the animals received an intragastric gavage of either sodium chloride (ctrl, n = 12) or ethanol (EtOH, n = 12) to simulate an AAI. Fx groups underwent osteotomy with the placement of an external fixator, and sham groups received only the external fixator. Twenty-four hours later, mice were euthanized, and liver sampling was performed. (**A**) Quantification of IL-10 protein (**B**) and *Il10* gene expression in liver tissue homogenates using mouse-specific ELISA kits and qRT-PCR, respectively. (**C**) Quantification of IL-6 protein (**D**) and *Il6* gene expression. (**E**) Protein concentration of MCP-1 is shown. The relative gene expression of (**F**) *Cxcl2*, (**G**) *Sost*, and (**H**) *Ctnnb1* normalized to *Gapdh* was calculated by using the comparative threshold-cycle 2^−DDCT^ method. * *p* < 0.05, n = 6 per group.

To investigate the impact of fracture and AAI on general hepatic inflammatory responses, both protein and gene expression levels of selected pro- and anti-inflammatory cytokines, as well as markers of the Wnt signaling, were evaluated (Figure 4).

The anti-inflammatory cytokine IL-10 was significantly reduced in both fracture groups Fx ctrl and Fx EtOH vs. the corresponding sham group (*p* < 0.05, Figure 4A). Gene expression of *Il10* was significantly reduced in the Fx EtOH group compared to all other experimental groups (*p* < 0.05, Figure 4B).

AAI resulted in significantly reduced protein levels of the pro-inflammatory cytokine IL-6 in the Fx EtOH group vs. Fx ctrl group (*p* < 0.05, Figure 4C). However, *Il6* gene expression remained unaffected by Fx or AAI (Figure 4D).

The protein levels of MCP-1, which are critical for the regulation of monocytes/macrophages migration and infiltration, were significantly reduced following fracture in both the Fx ctrl group as well as in the Fx EtOH group vs. the corresponding sham group (*p* < 0.05, Figure 4E). This reduction was further enhanced by AAI in the Fx EtOH group, which was significant compared to the Fx ctrl group (*p* < 0.05, Figure 4E).

Gene expression of *Cxcl2*, a chemokine primarily secreted by monocytes/macrophages during inflammatory responses, was significantly downregulated after fracture in both Fx ctrl and Fx EtOH vs. the corresponding sham group (*p* < 0.05, Figure 4F).

Evaluation of the Wnt signaling pathway, known for its regulatory role in post-traumatic inflammation [30], revealed that AAI significantly reduced the gene expression of sclerostin (*Sost*), a Wnt inhibitor, in the Fx EtOH group vs. Fx ctrl group (*p* < 0.05, Figure 4G). Concurrently, expression of β-catenin (*Ctnnb1*), a key activator in the Wnt signaling pathway, was significantly increased specifically in the Fx EtOH group vs. Fx ctrl group (*p* < 0.05, Figure 4H).

### 2.5. Fracture and AAI Modulate Wnt and NF-κB Signaling

**Figure 5:**
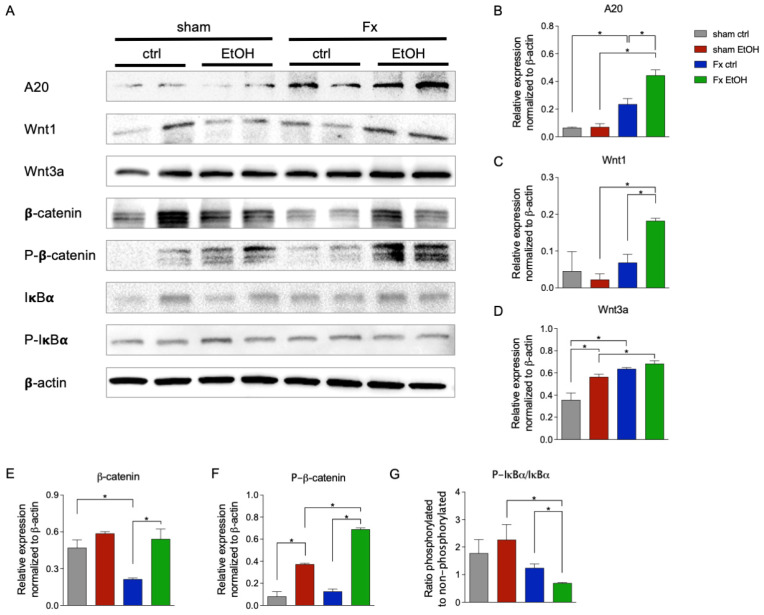
Regulation of Wnt and NF-κB signaling after acute alcohol intoxication (AAI) and fracture in the liver. Two hours before the initiation of experiments, the animals received an intragastric gavage of either sodium chloride (ctrl, n = 12) or ethanol (EtOH, n = 12) to simulate an AAI. Fx groups underwent osteotomy with the placement of an external fixator, and sham groups received only the external fixator. Twenty-four hours later, mice were euthanized, and liver sampling was performed. (**A**) Western blot analysis and quantification of protein expression levels of (**B**) A20, (**C**) Wnt1, (**D**) Wnt3a, (**E**) non-phosphorylated β-catenin, and (**F**) phosphorylated β-catenin, as well as (**G**) the ratio of phosphorylated and non-phosphorylated IκBα in liver tissue. The relative protein expression of normalized to β-actin was calculated and is shown. * *p* < 0.05, n = 6 per group.

To further elucidate the influence of fracture and AAI on the regulation of Wnt and NF-κB signaling pathways, Western blot analysis was conducted (Figure 5).

The ubiquitin-editing enzyme A20, a critical regulator of both pathways, demonstrated significantly increased protein levels in both Fx ctrl and in the Fx EtOH group compared to their respective sham group (*p* < 0.05, Figure 5A,B). Additionally, AAI further augmented A20 protein expression significantly in the Fx EtOH group vs. Fx ctrl group (*p* < 0.05, Figure 5A,B).

Western blot analysis also revealed a modulated activation of Wnt signaling in response to AAI. Wnt1 protein expression was significantly increased in the Fx EtOH group vs. corresponding sham EtOH group, as well as vs. the Fx ctrl group (*p* < 0.05, Figure 5A,C). Notably, Wnt3a expression was significantly upregulated by AAI, as shown by a significant increase in the sham EtOH group vs. sham ctrl group (*p* < 0.05, Figure 5A,D). Also, a significant fracture-induced Wnt3a increase in the Fx ctrl group vs. sham ctrl, and in the Fx EtOH group vs. sham EtOH, respectively, was observed (*p* < 0.05, Figure 5A,D). Examination of β-catenin protein levels showed a significant reduction after fracture in the Fx ctrl group vs. sham ctrl group (*p* < 0.05, Figure 5A,E), while such a reduction was not observed in the Fx EtOH group vs. sham EtOH group. β-catenin protein expression was significantly reduced in the Fx ctrl group vs. Fx EtOH group (*p* < 0.05, Figure 5A,E), suggesting that AAI in the Fx group reversed or prevented the fracture-induced β-catenin reduction. Furthermore, AAI significantly increased phosphorylated β-catenin levels in both sham EtOH group vs. sham ctrl group as well as in the Fx EtOH group vs. Fx ctrl group, respectively (*p* < 0.05, Figure 5 A,F). Also, phosphorylated β-catenin levels were significantly enhanced in the Fx EtOH group vs. both sham EtOH group and Fx ctrl group (*p* < 0.05, Figure 5 A,F).

NF-κB signaling activation was assessed by calculating the ratio of phosphorylated to non-phosphorylated IκBα. Results indicated a significant reduction in NF-κB activity in the Fx EtOH group vs. sham EtOH group (*p* < 0.05, Figure 5A,G). AAI significantly reduced the ratio of phosphorylated to non-phosphorylated IκBα in the Fx EtOH group vs. Fx ctrl group (*p* < 0.05, Figure 5A,G).

## 3. Discussion

In this study, we found that neither fracture alone nor fracture with AAI caused significant liver damage, as indicated by unchanged injury scores and caspase-3-positive apoptotic cell counts. However, fracture triggered a hepatic inflammatory response. characterized by increased PMNL infiltration and altered monocyte populations, which was further enhanced by AAI. Despite the absence of overt liver injury at the investigated time point, these findings suggest notable inflammatory and regenerative changes, supported by altered Wnt and NF-κB signaling pathway activity after fracture and AAI. Overall, our results indicate that fracture impacts liver inflammation and regeneration responses, effects amplified by AAI.

Despite the physical distance between bone and liver tissue, their interaction through systemic signaling molecules is well documented. Hepatic factors, so-called “hepatokines”, may directly impact the bone [31]. As an example, insulin-like growth factor (IGF)-1 plays an important role in bone homeostasis and growth, as well as in the maintenance of bone mass in adulthood [32]. Reduced IGF-1 levels, as seen in liver diseases like non-alcoholic fatty liver disease, contribute to osteoporosis [33,34,35]. Similarly, liver-derived fibroblast growth factor (FGF)21 promotes osteoclast activity and inhibits osteoblasts [36], further driving bone loss [37]. Conversely, bone-derived “osteokines” like FGF23 affect liver function, emphasizing the complexity of inter-organ communication [38]. Elevated systemic FGF23 levels are associated with poor liver disease prognosis [39], while the transforming growth factor (TGF)-β, also bone-derived, inhibits hepatocyte proliferation and promotes fibrosis [31,40].

Under normal conditions, bone and vasculature interact stably, but fractures disrupt bone, blood vessels, and surrounding tissues [3,4], triggering local and systemic inflammation essential for fracture healing [4,5,6]. Our results support the systemic impact of fracture trauma, consistent with clinical reports of elevated cytokines (IL-1β, IL-6, CXCL1) and neutrophilia post-fracture [9,10]. While systemic inflammatory response can affect distant organs from the injury site [41,42], our model showed that despite a present local inflammation, no significant liver injury 24 h post-fracture was present. In agreement with previous studies, we observed enhanced hepatic neutrophil infiltration and slightly elevated local CXCL1 levels, a chemokine known to recruit PMNL [10,43,44,45,46], indicating a modest but measurable hepatic inflammatory response after fracture.

While Kobbe et al. reported increased hepatic IL-6 levels after bilateral fracture [10], we did not observe this in our model, possibly due to differences in fracture severity [10]. However, we noted reduced IL-10 and MCP-1 levels in the liver, indicating a disturbed immune response. Given IL-10’s role in preventing acute liver failure [47] and MCP-1’s importance in activation and recruitment of monocytes [48,49], these changes may reflect early hepatic immune dysregulation post-fracture. Interestingly, despite increased neutrophil infiltration, flow cytometry showed a reduced overall neutrophil proportion, suggesting rapid turnover or selective recruitment. Additionally, fracture-induced alterations in monocyte polarization from pro-inflammatory (M1) toward anti-inflammatory (M2) phenotypes indicate an adaptive hepatic immunological shift, potentially facilitating resolution of inflammation or tissue repair [50]. This indicates that 24 h post-fracture, the healing process in the liver may have been initiated. Intriguingly, our analyses also revealed discrepancies between cytokine protein levels and their corresponding gene expression. Specifically, CXCL1 and IL-6 protein levels were increased post-fracture without corresponding mRNA changes, implying post-transcriptional or -translational mechanisms such as protein stabilization or degradation pathways. These findings suggest potential regulatory checkpoints in hepatic cytokine dynamics that require further investigation.

In contrast to the reduced hepatic IL-10 levels post-fracture, which indicate an impaired anti-inflammatory response, elevated liver A20 protein expression and reduced NF-κB activity suggest a compensatory anti-inflammatory regulatory mechanism following trauma. A20, a key negative regulator of NF-κB signaling [19], is essential for preventing excessive inflammation and early mortality in A20-deficient animals [51]. Its upregulation may explain the suppressed MCP-1 expression as observed in our experiments. Unlike previous multi-trauma models in combination with fracture, showing increased NF-κB activation [42,52], our findings likely reflect differences in trauma severity and type of injury. Notably, we also found increased A20 protein post-fracture, which additionally modulates Wnt signaling [20]. Furthermore, we analyzed the gene expression of further inflammatory markers and regulators of the Wnt signaling. CXCL2, a cytokine secreted by monocytes and neutrophils at the site of inflammation [53], was downregulated after femur fracture. In contrast, the expression of the regulator of the Wnt signaling, such as sclerostin (*Sost*) and β-catenin (*Ctnnb1*) [54,55], was not regulated after fracture compared to control animals. A novel aspect of our study is the significant modulation of Wnt signaling pathways observed post-fracture, evidenced by increased expression of Wnt pathway activators Wnt1 and Wnt3a [54]. The Wnt signaling is transmitted intracellularly via β-catenin, which is stabilized after activation and regulates nuclear gene expression [54]. However, despite elevated upstream Wnt proteins, β-catenin levels paradoxically decreased after fracture, potentially impairing downstream Wnt signaling essential for hepatic regeneration. The key role of Wnt signaling in liver regeneration was demonstrated in a two-thirds partial hepatectomy model before [56]. Thus, our results point to complex cross-talk between Wnt and NF-κB pathways, potentially mediated by A20.

AAI further complicated hepatic inflammatory responses. As with fracture alone, AAI did not cause overt liver damage. However, AAI prior to fracture significantly increased hepatic CXCL1 and IL-1β levels, enhancing neutrophil infiltration post-fracture. While neutrophil accumulation is typically linked to liver inflammation [57,58], here, the overall inflammatory response appeared partially suppressed, as indicated by reduced *Cxcl2*, IL-6, or MCP-1 levels. These findings align with previous preclinical studies showing attenuated hepatic inflammation following AAI [24,25,27,28].

These dual effects suggest that AAI may enhance early inflammatory cell recruitment while concurrently inducing immunosuppression, potentially via elevated A20 expression and resultant NF-κB suppression. A20 levels were twice as high after AAI compared to fracture alone, with reduced NF-κB activity [19], as shown by a decreased phosphorylated-to-non-phosphorylated ratio of IκBα. This indicates that A20 is regulated not only by inflammation but also by AAI, though the mechanism remains unclear. Interestingly, AAI also affected Wnt signaling, increasing the presence of activators of the Wnt pathway, Wnt1 and Wnt3a, compared to the fracture-alone group. Remarkably, AAI reversed fracture-induced reductions in β-catenin, fully restoring Wnt signaling. Yet, concurrent increase in the β-catenin phosphorylation by AAI suggests Wnt signaling pathway inhibition. Together, these findings highlight alcohol’s complex immunomodulatory effects post-trauma, simultaneously promoting and suppressing inflammation, with A20 potentially acting as a key mediator.

This study has several limitations. First, analyses were limited to a single early post-trauma time point (24 h), which may not capture the full dynamics of inflammatory and regenerative responses. Future studies should include multiple time points (both earlier and later) to fully characterize and assess the progression and resolution of liver responses post-fracture and AAI. Second, this investigation was limited to young male mice. Given known sex-specific differences in immune responses to trauma and alcohol intoxication [59,60], future studies should include female mice to determine sex-specific differences. Moreover, rising alcohol consumption among females further necessitates the inclusion of female subjects in future research. Additionally, the study exclusively utilized young mice; thus, findings may not adequately represent physiological responses in elderly populations who commonly experience fractures. Elderly individuals exhibit distinct immune and regenerative responses, highlighting the need for age-diverse study populations to enhance clinical relevance. Furthermore, observed discrepancies between cytokine protein and mRNA levels, as well as paradoxical responses such as increased neutrophil infiltration with decreased total neutrophil proportion, were not fully clarified mechanistically. Further studies employing additional molecular analyses are required to elucidate underlying regulatory mechanisms, such as post-transcriptional or translational regulation. While our findings suggest potential immunomodulatory and regenerative effects of AAI, interpretation should be cautious. Animal models, although valuable, do not directly replicate human responses; thus, extrapolation of these findings to clinical settings requires careful consideration and validation through clinical studies. Future investigations addressing these limitations are critical for fully elucidating the complexity of inflammatory and regenerative processes following trauma and the precise role alcohol plays in modulating these pathways.

In conclusion, this study demonstrates that isolated fracture induces hepatic inflammatory and regenerative changes, even in the absence of overt liver injury. Acute alcohol intoxication further modulates these responses through complex effects on cytokine expression, neutrophil infiltration, and key signaling pathways (NF-κB and Wnt). These findings highlight alcohol’s significant role in post-traumatic organ responses and underscore the need for further research to inform clinical management of trauma patients with alcohol intoxication.

## 4. Materials and Methods

### 4.1. Animal Care, Husbandry, and Group Organization

All animal experiments were performed in compliance with international regulations for the care and use of laboratory animals (ARRIVE guidelines and EU Directive 2010/63/EU for animal experiments) and were approved by the ethical committee of the Lower Saxony State Office for Consumer Protection and Food Safety (LAVES; No. 33.12-42502-17/2491). Twenty-four male C57BL/6J mice (Janvier Labs, Le Genest-Saint-Isle, France) with an age of 17–26 weeks (average 19.0 ± 1.7 weeks) were used for experiments. Fracture (Fx) was introduced as described before [61], while sham animals did not receive a fracture. Twelve mice received NaCl (ctrl), and 12 mice received an EtOH gavage. Experiments were initiated as soon as one week after acclimatization. All animals were housed in the central animal facility of the Hannover Medical School (MHH) under standardized conditions. Mice were randomly assigned to one of the four groups: sham ctrl, sham EtOH, Fx ctrl, and Fx EtOH, as described below (each group: n = 6).

### 4.2. Experimental Model

Two hours before trauma induction, the animals received a weight-dependent (12.66 µL/g bodyweight) intragastric gavage of either 0.9% NaCl or 35% EtOH (diluted in 0.9% NaCl). The alcohol concentration corresponds to 3.5 g/kg bodyweight [62]. Alcohol concentration was chosen based on previous studies, as it leads to fatty changes in the liver of the mice [63]. The volume was administered in two steps, each with half the total volume and a break of 15 min. A red-light lamp was positioned in front of the cages to prevent the animals from cooling down after gavage, even if they remained inactive or drowsy. Until the surgery, the animals were free to move, allowing them to regulate their proximity to the heat source as needed.

Prior to surgery, mice received a subcutaneous injection (s.c.) of carprofen (5 mg/kg bodyweight, Zoetis Inc., Parsippany, NJ, USA) and butorphanol (1 mg/kg body weight, Zoetic Inc. Parsippany, NJ, USA), and operation areas were locally anesthetized with prilocaine hydrochloride. Animals in the fracture group received a standardized osteotomy at the diaphysis of the right femur as described previously [64]. Briefly, under deep inhalation anesthesia using isoflurane (Baxter Deutschland GmbH, Unterschleißheim, Germany), a commercially available external fixator (MouseExFix simple L 100%, RISystem, Davos, Switzerland) was implanted into the right femoral bone of all animals. In the fracture group, an osteotomy gap of 0.5 mm was created, using a 0.44 mm Gigli wire saw at the mid-shaft of the femur at the diaphyseal part between the two middle pins (RISystem, Davos, Switzerland). For analgesia, animals received metamizole (200 mg/kg body weight) in the drinking water after surgery and an s.c. injection of carprofen and butorphanol according to indication.

### 4.3. Harvesting Procedures

Twenty-four hours later, the mice were euthanized by intraperitoneal injection of ketamine (75 mg/kg bodyweight, Zoetic Inc., Parsippany, NJ, USA) and medetomidine (1 mg/kg bodyweight, Zoetic Inc., Parsippany, NJ, USA). Blood was obtained via cardiac exsanguination, and sacrifice was finalized by cervical dislocation. The mice were perfused with phosphate-buffered saline (PBS) through the heart with a 21-gauge blunt-tipped syringe (BD, Franklin Lakes, NJ, USA). The left lateral liver lobe was ligated, where one part was snap-frozen in liquid nitrogen and stored at −80 °C until further use. The other part of the liver lobe was further preceded for flow cytometric evaluations. Afterwards, mice were perfused with 10 mL 4% buffered Zn-formalin solution (Thermo Fisher Scientific, Waltham, MA, USA) through the heart. The right liver lobe was removed and fixed for 24 h in 4% buffered formalin and transferred to 70% EtOH for long-term storage prior to histological analyses.

### 4.4. Examination of Liver Damage

Liver sections of 3 µm from the paraffin-embedded organs were prepared for hematoxylin–eosin (HE) and immune histological staining. After deparaffinization with Roti-Histol (Carl Roth, Karlsruhe, Germany), the sections were rehydrated and stained for 10 min at room temperature with Hemalum solution (Carl Roth, Karlsruhe, Germany). Afterwards, the slides were rinsed with water for 10 min and counterstained with eosin (Carl Roth, Karlsruhe, Germany) for 3 min. The slides were dehydrated by an ascending alcohol series and mounted (Mountex, Medite Medical GmbH, Burgdorf, Germany). Damage to the liver was assessed in a blinded method based on the following parameters: random necrosis, single-cell degeneration/necrosis or individualization, zonal necrosis (perivenous), vacuolization of its cells, and vacuolization of hepatocytes. Each parameter was scored with 0 (not observed), 1 (mild), 2 (moderate), or 3 (marked). The mean of all six values per group served as the liver injury score (LIS).

### 4.5. Immune Histological Staining of CXCL1, Neutrophil Elastase, and Active Caspase-3

Paraffin-embedded liver tissue sections were prepared for immunohistological staining. Briefly, the 3 µm sections were deparaffinized twice for 5 min using Roti-Histol and subsequently rehydrated through a series of ethanol dilutions (100%, 90%, and 70%). Epitope retrieval was carried out using R-Universal epitope recovery buffer (Aptum, Kassel, Germany) with the 2100-Retriever system (Prestige Medical, Blackburn, UK) at 121 °C for 20 min, following the manufacturer’s instructions. To inhibit endogenous peroxidase activity, sections were treated with hydrogen peroxide (Peroxidase UltraVision Block, Thermo Fisher Scientific, Waltham, MA, USA) for 20 min. Primary antibodies targeting C-X-C motif chemokine (CXCL)1, neutrophil elastase (NE), and active caspase-3 were used. All primary antibodies were diluted in Antibody Dilution Buffer (DakoCytomation, Carpinteria, CA, USA), as recommended by the manufacturer, and incubated for 1 h at room temperature: rabbit anti-mouse CXCL1 (1:300; #ab269939; Abcam, Cambridge, MA, USA), rabbit anti-mouse NE (1:200; #bs-6982R; Bioss, Woburn, MA, USA), and rabbit anti-mouse active caspase-3 (Asp175) (1:300; #9661; Cell Signaling Technology, Danvers, MA, USA). As a secondary antibody, goat anti-rabbit IgG-HRP (#414341F; Histofine Simple Stain Mouse MAX PO (R), Nichirei Biosciences Inc., Bellevue, WA, USA) was used and incubated for 30 min at room temperature. Specific binding was detected with 3-amino-9-ethylcarbazol (AEC, DCS Innovative Diagnostik-Systeme, Hamburg, Germany), and the sections were counterstained with hematoxylin (Carl Roth, Karlsruhe, Germany) and mounted using Medite Medical GmbH (Burgdorf, Germany). Images were obtained using an Axio Observer Z1 microscope (40× objective, Zeiss, Göttingen, Germany). Images were evaluated using ImageJ software 1.54p. Neutrophil elastase and caspase-3 positively stained cells were counted in 25 high-power fields at 400× magnification. CXCL1 staining was evaluated by determination of the mean intensity values.

### 4.6. Gene Expression Analysis

For RNA extraction, liver tissue was mechanically homogenized using the Precellys 24 Homogenizer (Bertin Technologies, Montigny-le-Bretonneux, France) in combination with the buffer provided in the RNeasy Mini Kit (Qiagen, Hilden, Germany). Total RNA was isolated according to the manufacturer’s protocol (Qiagen, Hilden, Germany). To eliminate any residual genomic DNA contamination, the samples were treated with the RNase-Free DNase Set (Qiagen, Hilden, Germany), as per the manufacturer’s guidelines. The quality and concentration of the extracted RNA were assessed using Tecan’s NanoQuant Plate on the Spark M10 Microplate Reader (Tecan, Männedorf, Switzerland). RNA integrity was verified by measuring the A260/A280 and A260/A230 absorbance ratios to ensure high-quality RNA for downstream applications. RNA was transcribed into cDNA using the iScript^TM^ cDNA synthesis Kit (BioRad, Hercules, CA, USA) following the manufacturer’s instructions. The synthesized cDNA was then used for quantitative real-time polymerase chain reaction (qRT-PCR) analysis. SYBR Green qPCR Master mix (BioRad, Hercules, CA, USA) was used for qRT-PCR in a total reaction volume of 25 μL and run in a CFX96 Touch Real-Time PCR Detection System (BioRad, Hercules, CA, USA). Primer specificity was verified through melting curve analysis, and no-template controls were included to detect possible contamination or non-specific amplification. *Cxcl1* (#qMmuCED0047655; BioRad, Hercules, CA, USA), *Il6* (#qMmuCID0005613; BioRad, Hercules, CA, USA), *Cxcl2* (#qMmuCED0050757; BioRad, Hercules, CA, USA), *Il10* (#qMmuCED0044967, BioRad, Hercules, CA, USA), *Sost* (#qMmuCED0045167; BioRad, Hercules, CA, USA), and *Ctnnb1* (#qMmuCID0006137; BioRad, Hercules, CA, USA) gene expression was analyzed relative to the housekeeping gene *Gapdh* (qMmuCED0027467; BioRad, Hercules, CA, USA) using the ΔΔCT method.

### 4.7. Protein Expression Levels via ELISA

Liver tissue was homogenized in lysis buffer (#FNN0021, Invitrogen, Waltham, MA, USA) at 4 °C and centrifuged at 20,000× *g* for 30 min at 4 °C to remove debris. The supernatants were collected and stored at −80 °C for further analyses. Tissue cytokine concentration of IL-1β, IL-10, IL-6, and monocyte chemoattractant protein (MCP)-1 were determined using mouse-specific ELISA kits (R&D Systems, Minneapolis, MN, USA) following the manufacturer’s protocol. Results were quantified using the Spark M10 microplate reader (Tecan, Männedorf, Switzerland).

### 4.8. Flow Cytometry

A 25 mg amount of one liver lobe was taken and processed by the Minute Single-Cell Isolation protocol (Invent Biotechnologies, Plymouth, MN, USA). A 40 μL volume of isolated and in FACS buffer resuspended single-cell pellets was stained for flow cytometric analysis, as described below. Samples were kept chilled on ice and incubated with the following antibodies: PerCP-labeled CD45 monoclonal antibody (EM-05, eBioscience™, #MA1-10234), APC-eFLOR780-labeled CD11b monoclonal antibody (M1/70, eBioscience™, #47-0112-82), Super Bright 600-labeled Ly-6G monoclonal antibody (1A8-Ly6g, eBioscience™, #63-9668-82), and eFluor450-labeled Ly-6C monoclonal antibody (HK1.4, eBioscience™, #48-5932-82). After incubating for 30 min in darkness, samples were washed with 2 mL FACS buffer at room temperature by centrifuging at 400× *g*. After removing the supernatant, the pellet was treated with 1 mL FACS Lysing Solution (BD Biosciences) and incubated for 10 min at room temperature. Subsequently, samples underwent centrifugation for 7 min at 400× *g*, followed by two additional washing steps, each with 2 mL FACS buffer. Finally, the cell pellet was resuspended in 200 µL FACS buffer and stored protected from light on ice until analyzed by flow cytometry.

Stained single-cell preparations were examined with a Thermo Fisher Attune NxT flow cytometer and corresponding software. Fluorescence intensity thresholds were determined based on the respective isotype control antibodies. At least 3.0 × 10^4^ cells were assessed per sample, except in a few cases with lower cell counts. Single cells were identified through forward and side scatter parameters. CD45-positive cells were selected and gated based on CD11b expression to define the CD45 + CD11b+ leukocyte population in the liver. Within this population, Ly6G-positive and -negative neutrophils were further classified based on their expression levels. Similarly, within the CD45 + CD11b+ leukocyte population in the liver, Ly6G-negative and Ly6C-positive monocytes were further classified based on their expression levels. Individual gating thresholds were set according to cellular expression intensity patterns illustrated in Figure 3. Subsequently, the proportions of cell phenotype subsets were quantified.

### 4.9. Western Blot

A 15 µg amount of liver protein lysate was separated on a 10% polyacrylamide sodium dodecyl sulfate gel and transferred onto a polyvinylidene difluoride membrane (PVDF, Thermo Fisher Scientific, Waltham, MA, USA). Membranes were blocked with 5% bovine serum albumin (#A9418-100G; Sigma-Aldrich; St. Louis, MO, USA) in TBS-T for 1 h at room temperature. Staining for A20 (TNFAIP3), Wnt1, Wnt3a, β-catenin, phosphorylated β-catenin, IκBα, and phosphorylated IκBα was performed with the following primary antibodies diluted in 5% BSA in TBS-T and incubated overnight at 4 °C: rabbit monoclonal anti-TNFAIP3 (1:1000; #ab92324; Abcam, Cambridge, MA, USA), rabbit polyclonal anti-Wnt1 (1:1000; #ab15251; Abcam, Cambridge, MA, USA), rabbit polyclonal anti-Wnt3a (1:1000; #ab28472, Abcam, Cambridge, MA, USA), rabbit polyclonal anti-β-catenin (1:1000, #AF1329; R&D Systems, Minneapolis, MN, USA), rabbit monoclonal anti-β-catenin phospho S675 (1:1000; #ab314450, Abcam, Cambridge, MA, USA), mouse monoclonal anti-IκBα (1:250; #ALX-804-209-C100; Enzo Life Sciences; Lörrach; Germany), and mouse monoclonal anti-IκBα phospho (1:250; #ALX-804-235-C100; Enzo Life Sciences, Farmingdale, NY, USA). Mouse monoclonal anti-β-actin antibody (1:1000; #MAB8929, R&D Systems, Minneapolis, MN, USA) was used as housekeeping control. Membranes were then incubated for 1 h at room temperature with the appropriate horseradish peroxidase (HRP)-conjugated secondary antibodies: goat anti-rabbit (1:3000; #7074P2) and horse anti-mouse (1:3000; #7076P2; both Cell Signaling, Danvers, MA, USA). Proteins were detected using 1 mL of Immobilon Forte Western HRP substrate (#WBLUF0100; Millipore; Burlington, VT, USA). The integrated density of the individual protein bands was determined by ImageJ software 1.54p, and protein expression levels were normalized to β-actin by densitometry.

### 4.10. Statistics

The results were displayed as a bar chart showing the mean ± standard error of the mean. Data were tested for normal distribution using the Shapiro–Wilk normality test. As the data were not normally distributed, a Mann–Whitney U test was performed. Values of *p* < 0.05 were considered statistically significant. Statistical analysis was performed using GraphPad Prism 10.0 software (GraphPad Software, La Jolla, CA, USA).

## Data Availability

Data are available upon reasonable request from the corresponding author.
